# Effects of Focused-Ultrasound-and-Microbubble-Induced Blood-Brain Barrier Disruption on Drug Transport under Liposome-Mediated Delivery in Brain Tumour: A Pilot Numerical Simulation Study

**DOI:** 10.3390/pharmaceutics12010069

**Published:** 2020-01-15

**Authors:** Wenbo Zhan

**Affiliations:** 1 School of Engineering, University of Aberdeen, Aberdeen AB24 3UE, UK; w.zhan@abdn.ac.uk; Tel.: +44-(0)1224-272511; 2 Department of Mechanical Engineering, Imperial College London, London SW7 2AZ, UK

**Keywords:** blood-brain barrier disruption, brain tumour, drug transport, focused ultrasound, liposome-mediated delivery, mathematical model

## Abstract

Focused ultrasound (FUS) coupled with microbubbles (MB) has been found to be a promising approach to disrupt the blood-brain barrier (BBB). However, how this disruption affects drug transport remains unclear. In this study, drug transport in combination therapy of liposomes and FUS-MB-induced BBB disruption (BBBD) was investigated based on a multiphysics model. A realistic 3D brain tumour model extracted from MR images was applied. The results demonstrated the advantage of liposomes compared to free doxorubicin injection in further improving treatment when the BBB is opened under the same delivery conditions using burst sonication. This improvement was mainly due to the BBBD-enhanced transvascular transport of free doxorubicin and the sustainable supply of the drug by long-circulating liposomes. Treatment efficacy can be improved in different ways. Disrupting the BBB simultaneously with liposome bolus injection enables more free drug molecules to cross the vessel wall, while prolonging the BBBD duration could accelerate liposome transvascular transport for more effective drug release. However, the drug release rate needs to be well controlled to balance the trade-off among drug release, transvascular exchange and elimination. The results obtained in this study could provide suggestions for the future optimisation of this FUS-MB–liposome combination therapy against brain cancer.

## 1. Introduction

Malignant glioma is highly invasive and aggressive, with a high mortality rate and short survival time [1]. Although a number of drugs have been developed with outstanding anticancer effectiveness shown in preclinical trials, their clinical performance for treating brain tumours remains disappointing. This could be attributed to the blood-brain barrier (BBB) [2], which is able to prevent over 98% of drugs from crossing the blood vessel wall in routine chemotherapy [3].

Focused ultrasound (FUS) coupled with systemically injected microbubbles (MB) has been found to be a promising approach by which to open the BBB [4]. Despite recovering gradually after the sonication ends [5], the temporary blood–brain barrier disruption (BBBD) can successfully enable intravenously administrated drugs to enter brain tumours for cell killing. This enhanced transvascular transport could be more significant for drugs like doxorubicin, to which the BBB is normally nearly impermeable [6,7]. Given that its clinical use is highly limited by serious adverse effects, especially cardiotoxicity, doxorubicin in a liposome-encapsulated form has been approved by the FDA as an alternative [8]. However, it is not clear how BBBD influences drug transport in liposome-mediated delivery, which can largely determine delivery outcomes and treatment efficacy.

Numerical simulation has become an effective way to study chemotherapy [9]. It has the advantage of being able to incorporate realistic tumour and drug properties to mimic multiple drug-delivery processes, which are difficult to observe directly in in vivo experiments. An initial mathematical model was set up to examine the roles of different intra-tumoural environments on the delivery of antibodies [10,11,12]. In subsequent developments, mathematical descriptions of more realistic and complex processes were incorporated to tailor the model to different delivery systems and strategies [13,14,15,16]. The delivery of doxorubicin under various delivery conditions has been studied extensively by means of numerical simulation [17,18,19,20], while the performance of free doxorubicin in combination with FUS-induced BBBD has been evaluated based on an idealised tumour model in 2D [21]. However, there is still a lack of modelling studies on liposome-mediated delivery coupled with FUS-MB-induced BBBD.

This simulation study aimed to examine the effects of FUS-MB-induced BBBD on drug transport in liposome-mediated drug delivery. A realistic 3D geometrical model of a brain tumour and its surrounding tissue was reconstructed from magnetic resonance (MR) images. The multiphysics model adopted incorporated key delivery processes including FUS- and MB-induced BBBD and its recovery; drug exchange among blood, tumour and normal tissues; drug convective and diffusive transport in the interstitial fluid flow; release from liposomes; drug physical degradation and metabolic reactions; binding with proteins and cell uptake; etc. The delivery outcomes, including the cytotoxicity to tumour cells and the risk of cardiotoxicity, were evaluated in terms of drug exposure over time in the brain tumour and blood circulatory system, respectively.

## 2. Materials and Methods

### 2.1. Mathematical Model

This mathematical model consisted of several submodules in order to describe the interlinked physiological and physicochemical processes involved in the drug delivery. These included the interstitial fluid flow across the entire brain and transport of liposomal and/or free drug among the blood circulatory system and different tissue compartments.

#### 2.1.1. Interstitial Fluid Flow

Microvasculature in solid tumours is elongated, dilated and tortuous, and its morphological characteristics can vary considerably with the tumour’s specific type and growth stage [22]. The distance between capillaries is around 33–98 μm, which is 2–3-fold lower than the dimension of both the tumour and its surrounding tissues [12]. Hence, a brain tumour and its surrounding tissue can be treated as porous media, where the Navier–Stokes equation is applicable to describe the incompressible, Newtonian interstitial fluid flow. The function of microvasculature can then be considered a source term in the mass equation [11], as follows.

(1) $$\nabla \cdot v=F_{b}$$

(2) $$\nabla \left(\rho vv\right)=-\nabla p_{i}+\mu \nabla^{2}v-\left(\frac{\mu }{\kappa }\right)v$$

where the velocity and pressure of interstitial fluid flow are represented by **v** and *p_i_*, respectively. *ρ* and *μ* denote the density and viscosity of interstitial fluid, respectively, and *κ* is the tissue’s Darcian permeability. Starling’s law is used to calibrate the flux of fluid loss from blood (*F_b_*).

(3) $$F_{b}=K_{b}\frac{S}{V}\left[p_{b}-p_{i}-\sigma_{T}\left(\pi_{b}-\pi_{i}\right)\right]$$

where *p_b_* is the blood pressure and *K_b_* is the hydraulic conductivity of the blood vessel wall. The vascular density is represented by *S/V*, which is defined as the area of blood vessel wall in the total tissue volume. *σ_T_* is the averaged osmotic reflection coefficient for proteins in blood. *π_b_* and *π_i_* are the osmotic pressures of blood and interstitial fluid, respectively.

#### 2.1.2. Direct Delivery of Free Doxorubicin

**Figure 1:**
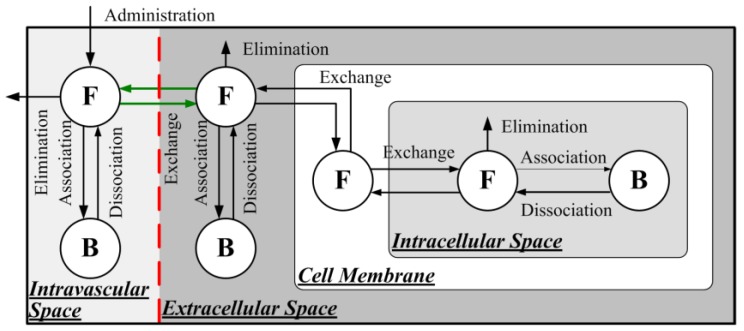
Transport processes involved in direct drug delivery in combination with focused-ultrasound-and-microbubble (FUS-MB)-induced blood–brain barrier disruption (BBBD). Red dashed line indicates the disrupted BBB, and the enhanced transvascular transport processes are highlighted in green.

Figure 1 shows the transport processes of non-encapsulated doxorubicin delivered via bolus injection. The doxorubicin concentration (C) in the intravascular space (IVS) can be described by

(4) $$C_{IVS}=\frac{Dose}{V_{F,d}}e^{-k_{F,c}t}$$

where Dose represents the total amount of the drug used in the treatment, and t is time. $V_{F,d}$ stands for the drug distribution volume, and $k_{F,c}$ is its plasma clearance rate. Free doxorubicin is able to associate and dissociate with proteins in a dynamic manner towards equilibrium [20]. Therefore, the intravascular concentration of free doxorubicin ($C_{F,IVS}$) can be calibrated by the mass conservation equation as follows.

(5) $$C_{IVS}=C_{F,IVS}+C_{B,IVS}=C_{F,IVS}\left(1+K_{IVS}\right)$$

where $C_{B,IVS}$ denotes the concentration of bound doxorubicin in blood and $K_{IVS}$ is the constant of drug binding with proteins.

Both the brain tumour and its surrounding tissue can be briefly divided into the intracellular space (ICS), cell membrane (CM), and extracellular space (ECS). Governed by the mass conservation equation, the free doxorubicin concentration in the entire tissue (*C_F_*) can be expressed in the form of [16,23]

(6) $$\frac{\partial C_{F}}{\partial t}=\upsilon_{ECS}D_{F,ECS}\nabla^{2}C_{F,ECS}-\nabla \cdot \left(\upsilon_{ECS}vC_{F,ECS}\right)+\upsilon_{ECS}Ex\left(C_{F,IVS},C_{F,ECS}\right)-\upsilon_{ECS}k_{F,e}C_{F,ECS}-\upsilon_{ICS}k_{F,e}C_{F,ICS}-\frac{\partial C_{B}}{\partial t}$$

where υ refers to the volume fraction of each tissue compartment and *k_F,e_* is the drug’s elimination rate due to the drug physical degradation and metabolic reactions.

The two-way exchange of free doxorubicin between IVS and ECS is determined by the convective transport with fluid loss from blood and diffusion driven by the transvascular concentration gradient.

(7) $$Ex\left(C_{F,IVS},C_{F,ECS}\right)=\left[F_{b}\left(1-\sigma_{F}\right)C_{F,IVS}+P_{F}\frac{S}{V}\left(C_{F,IVS}-C_{F,ECS}\right)\frac{{\mathrm{Pe}}_{F}}{e^{{\mathrm{Pe}}_{F}}-1}\right]$$

where $P_{F}\;$is the transvascular permeability of free doxorubicin, which is a function of time when the FUS-MB induced BBBD takes place. The Péclet number (${\mathrm{Pe}}_{F}$) is defined as ${\mathrm{Pe}}_{F}=\frac{F_{b}\left(1-\sigma_{F}\right)}{P_{F}S/V}$.

Two assumptions are further involved at this point: (I) the dynamic equilibrium of free doxorubicin concentration can be achieved in different tissue compartments [24] ($P_{ICS-ECS}=C_{F,ICS}/C_{F,ECS}$; $P_{CM-ECS}=C_{F,CM}/C_{F,ECS}$) and (II) the concentration of bound drug is linearly related to that of the drug in its free from [20] ($K_{ECS}=C_{B,ECS}/C_{F,ECS}$; $K_{ICS}=C_{B,ICS}/C_{F,ICS}$). Equation (6) can thus be rewritten as

(8) $$\frac{\partial C_{F,ECS}}{\partial t}=D_{F,ECS}^{*}\nabla^{2}C_{F,ECS}-v^{*}\cdot \nabla C_{F,ECS}-k_{F,e}^{*}C_{F,ECS}+Ex^{*}\left(C_{F,IVS},C_{F,ECS}\right)$$

where $v^{*}=\left(\upsilon_{ECS}/\omega \right)v$ is the apparent velocity of interstitial fluid flow, $D_{F,ECS}^{*}=\left(\upsilon_{ECS}/\omega \right)D_{F,ECS}$ is the apparent diffusion coefficient of free drug, $k_{F,e}^{*}=\left[\left(\upsilon_{ECS}+\upsilon_{ICS}\right)k_{F,e}+F_{b}\right]/\omega$ refers to the apparent drug elimination rate in tissue, $Ex^{*}\left(C_{F,IVS},C_{F,ECS}\right)=\upsilon_{ECS}Ex\left(C_{F,IVS},C_{F,ECS}\right)/\omega$ is the apparent drug exchange between IVS and ECS in both the brain tumour and normal tissue and ω is defined as $\omega =\upsilon_{ECS}\left(1+K_{ECS}\right)+\upsilon_{ICS}P_{ICS-ECS}\left(1+K_{ICS}\right)+\left(1-\upsilon_{ECS}-\upsilon_{ICS}\right)P_{CM-ECS}$.

#### 2.1.3. Delivery of Liposome-Encapsulated Doxorubicin

**Figure 2:**
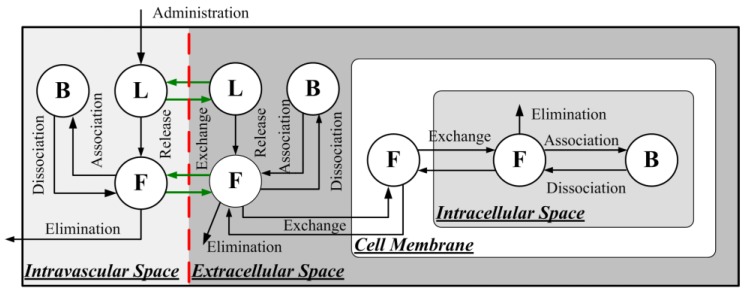
Drug transport in liposome-mediated delivery combined with FUS-MB-induced BBBD. The red dashed line refers to the disrupted BBB, and the enhanced transvascular transport is highlighted in green.

The drug transport in liposome-mediated delivery via intravenous administration is schematically shown in Figure 2. The pharmacokinetics of liposome-encapsulated doxorubicin ($C_{L,IVS}$) can be expressed as

(9) $$C_{L,IVS}=\frac{Dose}{V_{L,d}}e^{-\left(k_{L,c}+k_{rel}\right)t}$$

in which $V_{L,d}$ is the distribution volume of liposomes and $k_{L,c}$ is the plasma clearance rate. $k_{rel}$ denotes the drug release rate.

The extracellular concentration of liposomal doxorubicin ($C_{L,ECS}$) can be calculated by

(10) $$\frac{\partial C_{L,ECS}}{\partial t}=D_{L,ECS}\nabla^{2}C_{L,ECS}-\nabla \cdot \left(vC_{L,ECS}\right)-k_{rel}C_{L,ECS}+Ex\left(C_{L,IVS},C_{L,ECS}\right)$$

where $D_{L,ECS}$ is the diffusion coefficient of liposomes in tissue ECS. $Ex\left(C_{L,IVS},C_{L,ECS}\right)$ is defined in the same way as in Equation (7).

The intravascular concentration of free doxorubicin ($C_{F,\;IVS}$) is determined by the transvascular exchange between IVS and tissue ECS, drug release from liposomes, binding with proteins and plasma clearance.

(11) $$\frac{\partial C_{F,IVS}}{\partial t}=k_{rel}C_{L,IVS}-\frac{V_{tissue}}{V_{F,d}}Ex\left(C_{F,IVS},C_{F,ECS}\right)-k_{F,c}C_{F,IVS}-\frac{\partial C_{B,IVS}}{\partial t}$$

where $V_{tissue}$ is the volume of either brain tumour or its surrounding tissue. The extracellular concentration of free doxorubicin ($C_{F,ECS}$) is governed by

(12) $$\frac{\partial C_{F,ECS}}{\partial t}=D_{F,ECS}^{*}\nabla^{2}C_{F,ECS}-v^{*}\cdot \nabla C_{F,ECS}-k_{F,e}^{*}C_{F,ECS}+Ex^{*}\left(C_{F,IVS},C_{F,ECS}\right)+k_{rel}^{*}C_{L,ECS}$$

where $k_{rel}^{*}=k_{rel}/\omega$ refers to the apparent drug release rate from liposomes.

### 2.2. Model Geometry

**Figure 3:**
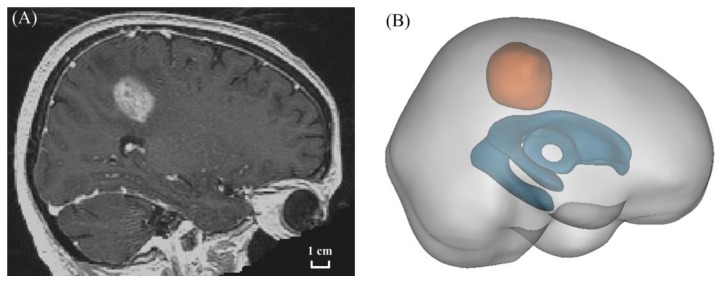
Model geometry. (**A**) MR image, and (**B**) reconstructed 3D geometry of brain tumour (orange) and its surrounding normal tissue (grey). The brain ventricle is coloured in cyan.

The 3D geometrical model of a brain tumour and surrounding normal tissue was reconstructed from anonymous MR images, which were acquired in three orthogonal planes. These images were stored on the image database TCIA, and are available for scientific purposes under a Creative Commons Attribution 3.0 Unported License [25,26]. Each image slice was 1 mm thick, and comprised 256 × 256 pixels. The dimension of each pixel was also 1 mm. Figure 3A shows a representative image slice as used in this study.

The brain tumour, ventricle and normal tissue were segmented based on the local signal intensity on each image slice using MIMICS (Materialise HQ, Leuven, Belgium). After being smoothed, these reconstructed 3D surfaces were imported into ANSYS ICEM CFD (ANSYS Inc., Canonsburg, PA, USA) to generate the computational mesh. The final volumetric mesh was composed of 4.6 million tetrahedral elements, which were tested to be fine enough to eliminate the grid-quality dependence. The 3D model geometry is shown in Figure 3B, where the volume of the brain tumour and its surrounding tissue were 2.47 × 10^−5^ m^3^ and 1.39 × 10^−3^ m^3^, respectively.

### 2.3. Model Parameters

Given that the simulation time window was much shorter than that of tumour growth, the biological and geometrical properties of brain tumour and normal tissue, as well as the transport properties of anticancer agents, were treated as being independent of time [17]. The baseline value of each parameter with respect to its category is listed in Table 1 and Table 2. In contrast to doxorubicin, which is unable to penetrate the BBB alone [6,7], surface modification with certain ligands successfully improves the liposome transvascular transport in brain tumour [27,28]. Therefore, the transvascular permeability of doxorubicin was assumed to be zero without BBBD, while the innate permeability of liposomes was set as 3.4 × 10^−9^ m/s [29] in the brain tumour.

Drug transvascular permeation upon BBBD can be described by an exponential decay function [21] as follows.

(13) $$P\left(t\right)=\{\begin{array}{cc} P_{0}+P_{s} & t<T_{s}\\ P_{0}+P_{s}\exp\left[-k_{r}\left(t-T_{s}\right)\right] & t\geq T_{s} \end{array}$$

where $P_{0}$ is the drug instinct transvascular permeability and $P_{s}$ refers to the enhancement due to the BBBD. $T_{s}$ is the sonication duration. For the small molecular drugs like free doxorubicin, the enhanced permeability correlates to its molecular weight (MW) [21]:

(14) $$P_{MW}/P_{\mathrm{Gd}-\mathrm{DPTA}}=1-0.5\mathrm{lg}\left(MW\right)$$

in which $P_{\mathrm{Gd}-\mathrm{DPTA}}$ is the transvascular permeability of Gd-DPTA, which was measured to be 2.0 × 10^−6^ m/s [30] when 0.6 MPa FUS was applied. Under similar sonication conditions, the transvascular permeability of 120 nm liposomes was about 4.25 times higher than the baseline value [31]. $k_{r}$ stands for the BBBD recovery rate, which can be calibrated using the semi-empirical formula [32]

(15) $$k_{r}=ln2\left(1+0.21d_{H}^{2}\right)/2.34\times 10^{4}$$

where $d_{H}$ is the hydrodynamic diameter (in nm) of the anticancer agents, and can be predicted based on the Einstein–Stokes equation [32].

### 2.4. Numerical Methods

The mathematical model was implemented in ANSYS FLUENT (ANSYS Inc., Canonsburg, PA, USA) for numerical solutions. The predicted pressure and velocity correction were correlated by the SIMPLEC algorithm. The second-order implicit Euler scheme and second-order UPWIND scheme were employed to achieve temporal and spatial discretisation of governing equations, respectively. The residual tolerance was set as 1 × 10^−5^ to control the simulation convergence, and the time step was fixed at 10 s to achieve the time-step-independent solutions. The governing equations for interstitial fluid flow were solved first to generate the hydraulic environment in the steady state. The obtained pressure and velocity were then imported into the submodules of drug transport at time zero to predict the drug delivery processes [44,45,46]. Drug concentration was assumed to be zero throughout the whole domain at the beginning of treatment.

### 2.5. Boundary Conditions

The gauge pressure on the brain surface and ventricle were specified as 658 Pa [47] and 1447 Pa [43], respectively, with zero flux of drug. The continuity condition [17] was applied at the interface between the tumour and normal tissue.

### 2.6. Quantification of Delivery Outcomes

The drug bioavailability for anticancer effectiveness and risk of cardiotoxicity was measured as the drug exposure over time (AUC), which is defined as

(16) $$AUC_{T}=\int_{0}^{T}C\left(t\right)dt$$

where the T is the considered period of treatment.

## 3. Results

### 3.1. Interstitial Fluid Flow

As the drug convective and diffusive transport in tissue ECS are both dependent on the interstitial fluid, its flow field was expected to play an important role in determining the delivery outcomes. In this study, the interstitial fluid flow was predicted by solving the governing Equations (1)–(3) throughout the entire brain, subject to the biological properties and the boundary conditions described above.

**Figure 4:**
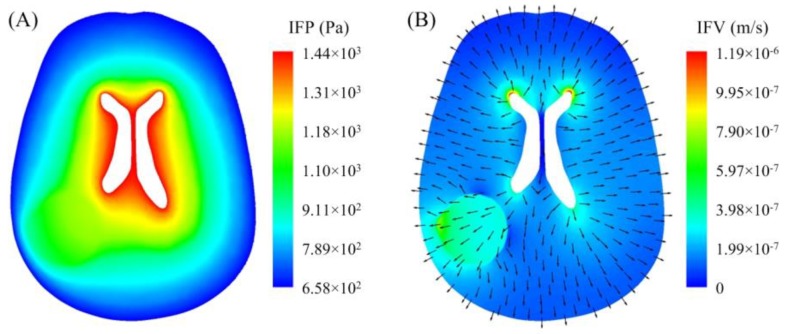
Predicted (**A**) interstitial fluid pressure and (**B**) velocity in the brain. Results are shown on a cross-section that covers regions of brain tumour, normal brain tissue and ventricle.

The spatial distribution of interstitial fluid pressure (IFP) on a brain cross-section is shown in Figure 4A. IFP reduced gradually from the ventricle to brain surface. However, this pressure was higher in the tumour, as shown in Table 3. This was attributed to the variation of microvasculature in tumour tissue; on one hand, the vasculature surface is enlarged, since the microvasculature becomes tortuous and elongated. On the other hand, large pores on vessel surfaces can significantly increase the hydraulic conductivity of the blood vessels, enhancing fluid leakage into the tumour ECS.

As shown in Figure 4B, the interstitial fluid flows across the entire brain from ventricle to brain surface, driven by the pressure gradient in the same direction. The comparisons presented in Table 3 denote that the interstitial fluid flow was faster in the brain tumour. This was due to the advanced fluid loss from blood [48] and the high hydraulic conductivity of the tumour tissue [49]. As a result, the drug convective transport was more effective in tumour. It is worth noting that the interstitial fluid velocity (IFV) was not uniform in the tumour. Due to the large pressure difference between the tumour and the brain surface, the velocity was higher in the tumour region, which was more superficial.

### 3.2. Baseline Study of Drug Transport and Accumulation

A total dose of 50 mg/m^2^ liposomal doxorubicin was administrated into a 70 kg patient’s circulatory system by bolus injection [17]. The FUS sonication and MB injection were supposed to start simultaneously with the chemotherapy, and to last for seconds [50,51,52]. This sonication duration was negligible, as it was short compared to the examined treatment duration of 24 h [21].

**Figure 5:**
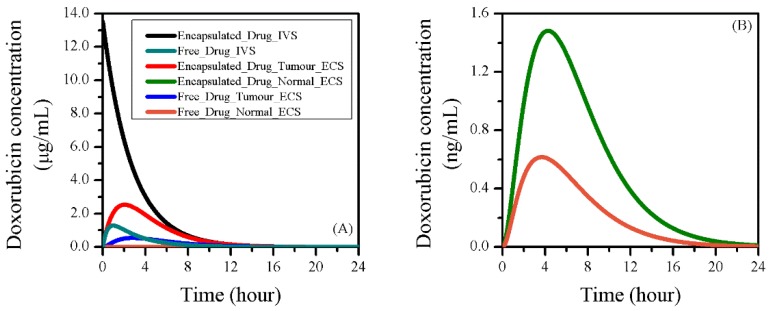
The concentration of doxorubicin in different forms in (**A**) each compartment of the brain tumour and the surrounding normal tissue. A close look in normal tissue is given in (**B**).

Figure 5 shows the predicted doxorubicin concentrations in each tissue compartment as a function of time. As the liposomes were injected into the blood stream over a very short duration (bolus injection), the intravascular concentration of liposomal doxorubicin concentration ($C_{L,IVS})$ peaked when the treatment started and decreased exponentially over time. Free doxorubicin concentration in IVS ($C_{F,IVS})$ continued to increase in the first 1.5 h as a result of the continuous drug release from the liposomes. This was followed by a gradual decline owing to the decrease of $C_{L,IVS}$ and the continuous drug plasma clearance. Liposomal doxorubicin continued to accumulate in tumour ECS until the 2 h point, driven by the transvascular concentration gradient. However, due to the reduction of this gradient and drug release, less doxorubicin could remain in the encapsulated form in tumour ECS as time went on. Since local drug release and the exchange with IVS were the two sources for free doxorubicin to accumulate in the tumour ECS, $C_{F,ECS}$ reached its peak around 3.5 h after the treatment started, with a time delay of 2 h compared to $C_{F,IVS}$.

A close look at the doxorubicin concentration in normal tissue is given in Figure 5B, where the drug presented similar trends as in the tumour ECS. However, the concentrations were about three orders of magnitude lower. This is because the drug reached the normal tissue from the tumour by convective and diffusive transport.

### 3.3. Comparisons to other Delivery Modes

**Figure 6:**
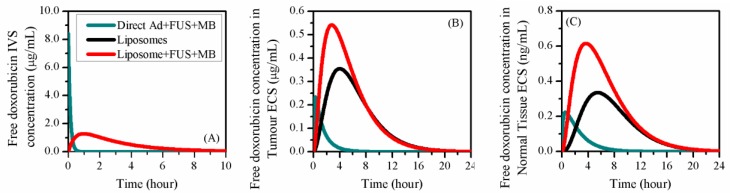
The time course of free doxorubicin concentration under different delivery modes. (**A**) IVS, (**B**) tumour ECS and (**C**) normal tissue ECS.

The delivery outcomes are compared to those of two control studies using the same dose and administration method in Figure 6. These control studies were specified as (I) direct delivery of free doxorubicin with BBBD, and (II) liposome-mediated delivery of doxorubicin without BBBD.

When the drug was directly administered in its free form, $C_{F,IVS}\;$reached its peak at the beginning of treatment and decreased sharply to zero in about 1 h. In contrast, liposome-mediated delivery effectively reduced the drug clearance via the blood, resulting in a gradual change of $C_{F,IVS}$ over time. Since BBBD does not affect the pharmacokinetics of liposomes and the dynamics of drug release, $C_{F,IVS}$ presented a similar time courses to liposome-mediated delivery both with and without BBBD.

**Figure 7:**
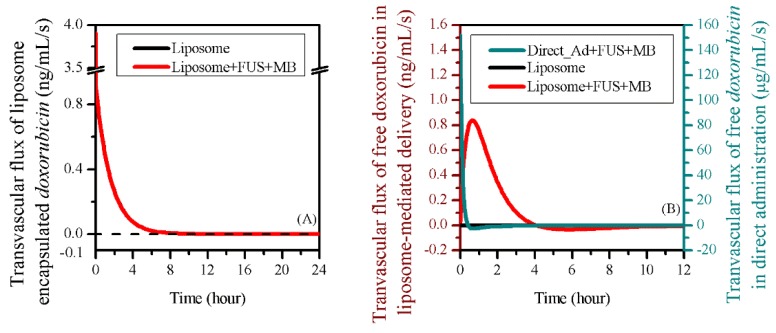
The transvascular flux of (**A**) liposome-encapsulated doxorubicin and (**B**) free doxorubicin between IVS and ECS in the brain tumour.

Free doxorubicin concentration in tumour ECS ($C_{F,ECS}$) was strongly dependent on the delivery mode. Although BBBD successfully enabled doxorubicin to cross the blood vessel wall, the drug accumulation was less effective in the treatment where free doxorubicin was directly administrated. This can be attributed to the fast decreases of IVS concentration and drug transvascular transport, as shown in Figure 6A and Figure 7B, respectively. In contrast, the combination of BBBD and liposome-mediated delivery significantly improve the drug accumulation in tumour tissue. The drug concentration in normal tissue showed similar trends as in tumour ECS; however, the magnitude is three orders lower.

The impacts of BBBD on the drug transvascular flux are shown in Figure 7. The comparisons indicated that the transvascular transport of liposome-encapsulated doxorubicin was less sensitive to BBBD. This was due to the fast BBBD recovery, for which half-life time was 7.74 s, as predicted by Equation (15) from Reference [32].

BBBD greatly affected the transport of free doxorubicin across the vessel wall. The flux under direct injection was three orders of magnitude higher. However, it decreased to negative in about 30 min, implying that the free doxorubicin began to be transported back from tumour ECS to IVS as the concentration gradient reversed, so that more drug was lost from the tumour ECS. In contrast, free doxorubicin in liposome-mediated delivery continued to pass through the vessel wall to the tumour ECS for 4 h. The drug accumulation in tumour ECS was therefore improved, as shown in Figure 6B. It is worth noting that the transvascular flux of free doxorubicin remained zero throughout the entire treatment when using liposomes alone. This is because no free doxorubicin could cross the blood vessel wall without BBBD.

The outcomes of the different delivery modes are compared in Table 4 in terms of the drug exposure over time (*AUC*). It is defined as the area under the curve of free drug concentration against time. The comparisons show that the combination of BBBD and liposome-mediated delivery successfully improved the bioavailability of doxorubicin in each compartment of the brain. Although more effective drug exposure in tumours could improve the drug’s anticancer efficacy, additional attention is required as the increased drug concentration in the blood circulatory system and normal brain tissue could raise the risk of adverse effects.

### 3.4. Impact of Release Rate

As the representative value of the time scale on which the liposomes released the loaded drug, release rate directly determines the toxicity and anticancer activity of a drug-delivery system [53,54,55]. It can vary across a wide range, depending on several factors such as the liposome formulation, fabrication approach, surrounding environment [56,57], etc. Sustainable release over weeks can be achieved using stealth liposomes [58], while temperature-sensitive liposomes are designed to release their loads in seconds to minutes [59]. Therefore, the release rate was changed within the range from 1 × 10^−6^·s^−1^ to 1 × 10^−^^3^·s^−1^ in this study.

**Figure 8:**
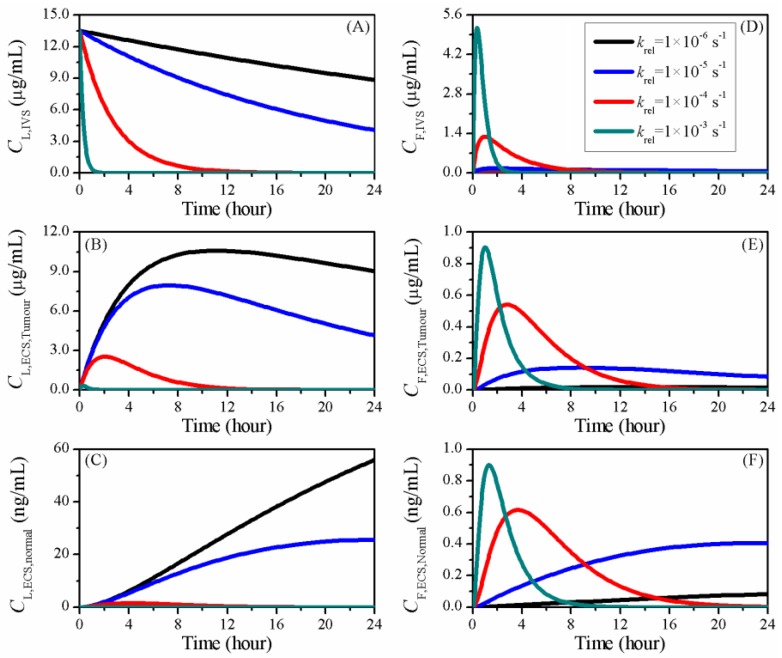
The time courses of doxorubicin concentration in treatments using liposomes with different release rates. Liposome-encapsulated doxorubicin in (**A**) IVS, (**B**) tumour and (**C**) normal tissue ECS; free doxorubicin in (**D**) IVS, (**E**) tumour and (**F**) normal tissue ECS.

The impacts of the release rate on doxorubicin concentrations are shown in Figure 8. It is not surprising that a slow drug release kept more doxorubicin in the encapsulated form in the blood, and thereby was able to provide a sustainable drug supply. A similar response was found for the liposome concentration in tumour ECS ($C_{L,ECS}$). This is because, on one hand, a high $C_{L,IVS}$ enabled the liposome-encapsulated drug to enter the tumour ECS in a continuous manner; on the other hand, the reduced $k_{rel}$ slowed down the drug release in tumour tissue. The $C_{L,ECS}$ in normal tissue had a similar sensitivity to the release rate, but it was orders of magnitude lower than in tumour tissue.

Results also showed that reducing the release rate could effectively lower the concentration peak of free doxorubicin, and lead to a more gradual variation of drug concentration with time. Although fast release could sharply raise the amount of free doxorubicin in a short period time, more drug was cleared out of the tumour due to the high elimination rates in both blood and tissue ECS, as shown in Table 1. Moreover, since there was no longer enough drug being released from liposomes, the concentration of free doxorubicin dropped quickly to a low level.

**Figure 9:**
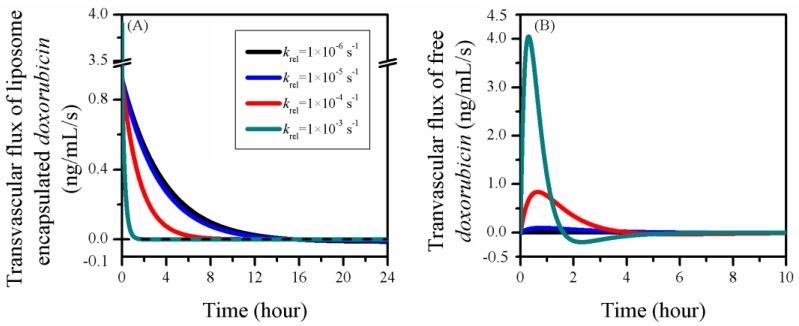
The time courses of drug transvascular flux in the treatments using liposomes with different release rates. (**A**) Liposome-encapsulated doxorubicin and (**B**) free doxorubicin.

The transvascular fluxes of doxorubicin in the treatment using different liposomes are plotted in Figure 9. Owing to the reduced availability, less liposomal drug could cross the blood vessel wall when the release rate was increased. In contrast, the transvascular flux of free doxorubicin became more volatile with the increase of release rate. As more free drug was released, the high $k_{rel}$ could significantly increase the transvascular concentration gradient after the treatment started. Consequentially, large amounts of free drug were transported into the tumour ECS. However, due to the fast decrease of $C_{F,IVS}$, as shown in Figure 8D, this transvascular flux fell quickly to negative when the free doxorubicin concentration in blood became lower than in tumour ECS. The transvascular flux gradually restored as time proceeded, as drug was slowly transported back to the blood.

The delivery outcomes using liposomes with different release rates are compared in Table 5. The availability of free drug in the IVS increased with the release rate, whereas the drug exposure in tissue ECS was non-linearly correlated to the release rate in the examined period. This finding indicates that the drug release rate can be optimised to maximise the treatment efficacy while maintaining a similar risk of side effects to the cardiovascular system.

### 3.5. Impact of BBBD Timing

**Figure 10:**
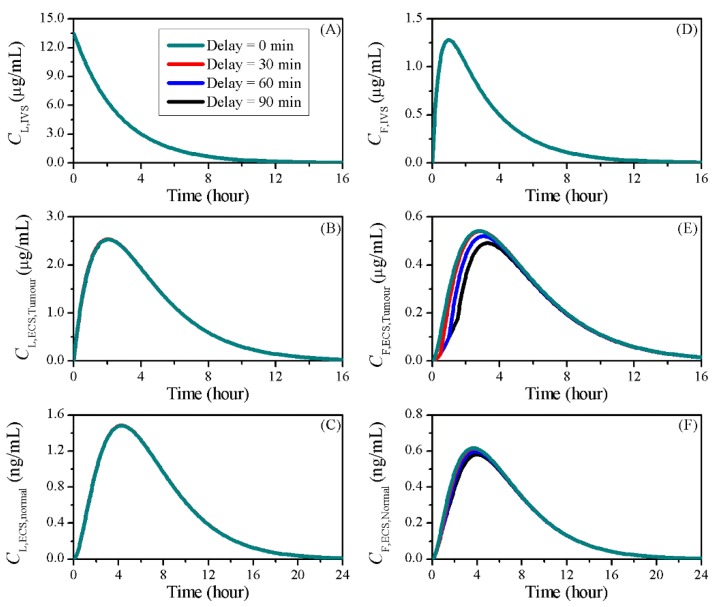
The time courses of doxorubicin concentrations in treatments with different BBBD timing. Liposome-encapsulated doxorubicin in (**A**) IVS, (**B**) tumour and (**C**) normal tissue ECS; free doxorubicin in (**D**) IVS, (**E**) tumour and (**F**) normal tissue ECS.

The starting time point of BBBD is a factor that can be well controlled in clinical operations. The BBB was disrupted simultaneously with the liposome injection in the baseline study. The delivery outcomes were compared to those of treatments in which BBBD was induced at 30, 60 and 90 min after the chemotherapy started, as shown in Figure 10. Given that the BBBD has no effect on liposome pharmacokinetics and release dynamics, identical time courses of $C_{L,IVS}$ and $C_{F,IVS}$ were found in each treatment. Although the liposomes presented similar concentration profiles in the brain tumour, less free doxorubicin was available for cell killing when the BBB opening was postponed. This was the same in normal tissue, since all the drug came from tumour ECS by convection and diffusion.

**Figure 11:**
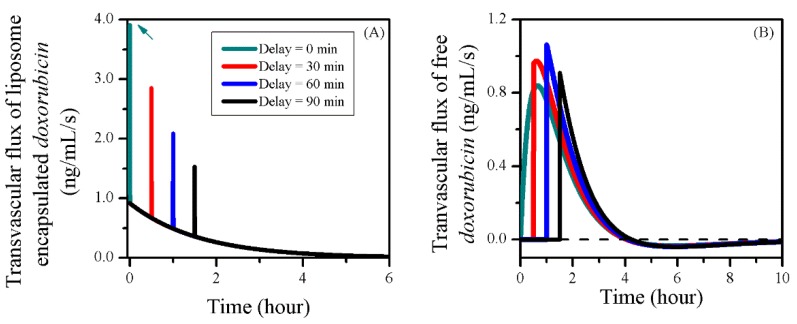
The time courses of drug transvascular flux in the treatments with different BBBD timings. (**A**) Liposome-encapsulated doxorubicin and (**B**) free doxorubicin.

The transvascular flux in treatments with different sonication timings is shown in Figure 11. Results showed that the transvascular flux of liposomes was reduced by postponing the BBBD. However, since the enhanced *P_L_* dropped quickly to its normal level, changing the BBBD timing had no obvious impact on the liposome ECS concentration in tumour tissue, as shown in Figure 10B.

This was different from free doxorubicin—its transvascular flux jumped to a higher peak during BBBD; however, it must be noted that there was no exchange of free doxorubicin between blood and tumour tissue before the BBBD occurred. As a result, the transvascular flux over the entire treatment period was low, and the free doxorubicin accumulation in tumour tissue was therefore reduced, as shown in Figure 10E.

The delivery outcomes compared in Table 6 show that for liposome-mediated delivery by bolus injection, reduction of anticancer effectiveness could be introduced when BBBD is postponed. However, the risk of adverse effects to the cardiovascular system remain similar.

### 3.6. Impact of Sonication Duration

The time window of FUS sonication is another controllable factor in clinical settings. It usually lasts for seconds in preclinical trials [50,51,52], whereas a 40 min sonication was applied in a previous in vivo experiment to increase doxorubicin delivery [60]. Hence, the delivery with FUS sonication for 15, 30 and 45 min was compared to the baseline study to examine the effects of this factor.

**Figure 12:**
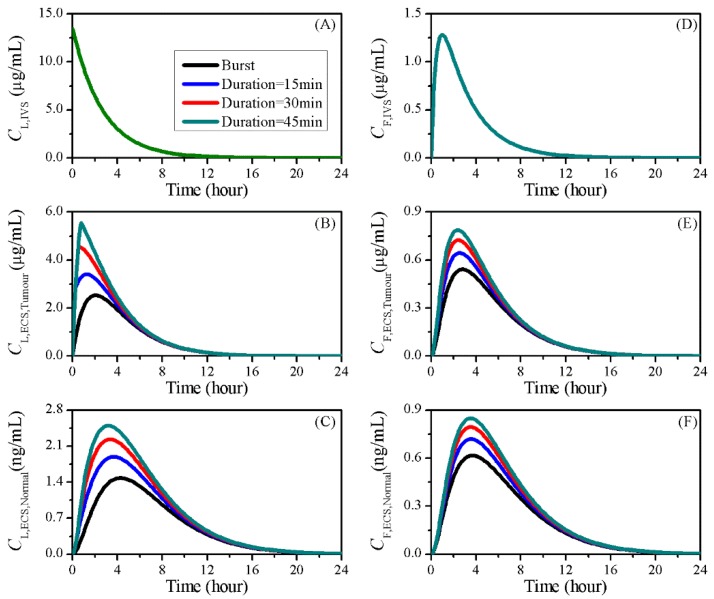
The time courses of doxorubicin concentrations in treatments with different sonication durations. Liposome-encapsulated doxorubicin in (**A**) IVS, (**B**) tumour and (**C**) normal tissue ECS; free doxorubicin in (**D**) IVS, (**E**) tumour and (**F**) normal tissue ECS.

The doxorubicin concentration in treatments with different sonication durations are shown in Figure 12. Given that BBBD has no impact on liposome transport within blood, the encapsulated doxorubicin presented the same time course for IVS concentration in different treatments. Similar trends were found for free doxorubicin IVS concentration, as this mainly determined by local drug release and plasma clearance which are both independent of BBBD. In contrast, the drug concentrations in tumour and normal tissue ECS were increased by prolonging the FUS sonication.

**Figure 13:**
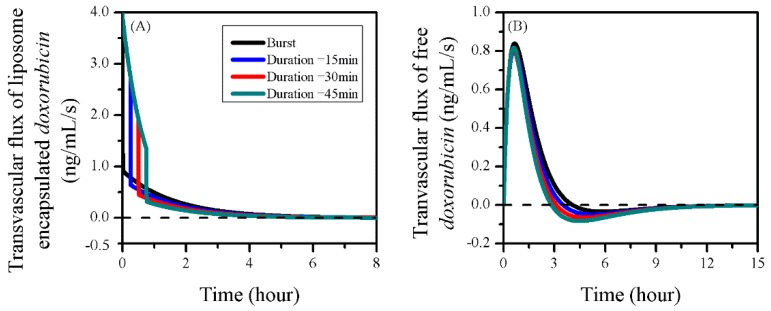
The time courses of drug transvascular flux in the treatments with different sonication durations. (**A**) Liposome-encapsulated doxorubicin and (**B**) free doxorubicin.

The impacts of sonication duration on the drug transvascular flux are given in Figure 13 as a function of time. Results showed that the IVS–ECS exchange of liposomal doxorubicin began to decline after the treatment started. However, this decline could be effectively slowed down by prolonging the FUS sonication, due to the enhanced liposome transvascular permeability. Consequentially, more liposomes were able to accumulate in the tumour ECS, as shown in Figure 12B. A sharp fall was observed after the sonication ended, because of the fast recovery of BBBD to liposomes [32].

Prolonging the sonication duration had limited impact on the gain of free drug from the blood, but resulted in the increase of drug loss by blood drainage. This is because the BBBD-enhanced $P_{F}$ remained at a higher level for free doxorubicin transport back to the blood. As a result, the improved drug release from liposomes was assumed to be the main reason for the effective free drug accumulation in tumour ECS shown in Figure 12E.

Table 7 compares the delivery outcomes of treatments with different sonication durations. Results showed that the anticancer efficacy could be successfully improved by increasing the FUS functioning time window. Simultaneously, the risk of adverse effects to brain normal tissue could also be increased. However, the similar drug availability in blood indicates that the sonication duration would have limited effects on the risk of cardiotoxicity.

## 4. Discussion

FUS and MB can successfully open the BBB, and thereby enable doxorubicin accumulation in tumour ECS for treatment. Modelling predictions further demonstrated that liposome-encapsulated drugs could effectively improve the delivery outcomes of combination therapy with FUS and MB. Owing to the fast recovery of BBBD to liposomes, burst FUS sonication had very limited impact on the transvascular transport of liposomal drug, as shown in Figure 7A. Therefore, the advantage of the FUS-MB-liposome combined delivery was mainly due to the improved transvascular permeability of free doxorubicin and sustainable drug supply by the long-circulating liposomes.

In order to examine the impact of enhanced liposome transvascular permeability on the delivery outcomes, the sonication duration was prolonged up to 45 min. Although the loss of free doxorubicin by capillary drainage was slightly raised, as shown in Figure 13, the modelling results showed that keeping the BBB open for a longer time effectively improved the accumulation of both the liposome-encapsulated and free doxorubicin, which could lead to better therapy. However, it is important to note that the enhancement of drug transvascular permeability is very limited when FUS is applied in isolation [31]. Therefore, the pharmacokinetics of MB must be considered in treatment design in order to achieve continuous BBBD. Since sonication has been generally performed for few seconds in preclinical trials, how to prolong FUS sonication and MB circulation for continuous BBBD needs to be explored in both experiments and simulations in the future.

Serval factors influence BBBD, including the frequency and power of FUS [61,62,63], sonication schedule [21,51], pharmacokinetics and dimensions of MB [64,65,66], molecular weight or size of the delivered agents [29,67], the biological properties of the microvasculature, etc. Despite several in vivo experiments having been carried out under different conditions [50,68,69,70], there is still a lack of a comprehensive mathematical model with which to describe the dependence of the permeability enhancement on the aforementioned key factors. Therefore, parameter studies were not performed in this pilot study to understand their effects. Extensive experimental data are required to establish a model for optimisation of this treatment design.

The treatment efficacy was found to be non-linearly correlated to the drug release rate. On one hand, the fast release enabled more drug to be released, achieving a high concentration in a short period of time. However, as shown in Table 1, the elimination and plasma clearance rate of free drug was orders of magnitude higher than when using liposomes. Consequently, the drug concentration reduced quickly to zero, leading to a low drug exposure over time in the tumour. On the other hand, the slow release could theoretically lead to a sustainable supply of free drug. It is still important to note that liposomes are continuously washed out by plasma clearance. As a result, drugs are highly likely to be cleared out of the body before even being released. Therefore, the release rate needs to be optimised to maximise anticancer effectiveness by maintaining a balance among drug release, transvascular exchange and elimination. A general liposome with the same release rate in IVS and ECS was applied in this study. However, this rate could be different depending on the intratumoural environment. For instance, drug release from pH-sensitive liposomes could be much quicker in tumour ECS than in blood, as acidity of the environments differs [71]. Thermosensitive liposomes are designed to be stable at body temperature, while a burst drug release can be achieved when the environmental temperature is above the pre-designed threshold [56,59]. Subsequent studies could therefore focus on the relationship between release rate and intratumoural environment in order to provide suggestions for improvement of the liposome properties.

The BBB can be permeabilised using FUS in a non-invasive, reversible manner. With the guidance of transcranial MR imaging, the targeting accuracy of FUS can be further improved to achieve localised treatment [72]. In practice, ultrasound contrast agents in the form of microbubbles are intravenously administrated as a first step. This is followed by the projection of FUS into the designed region of the brain. Triggered by the ultrasound, the microbubbles become activated to produce a range of chemical, mechanical and thermal effects that can transiently disrupt the tight junctions of the endothelial cells on the blood vessel wall [73]. The ability of this BBBD to improve drug delivery has been reported in terms of animal experiments in literature [74]. Moreover, there has been no significant brain tissue damage found in clinical trials [75], demonstrating the accuracy and safety of this combination drug-delivery strategy.

A multiphysics model was employed in this study to examine the effects of FUS- and MB-induced BBBD on drug transport in chemotherapy. The IFV was predicted as 0.43 and 0.13 µm/s in the brain tumour and its surrounding tissue, respectively, values which are both well within the range of 0.1 to 1.0 µm/s measured previously in in vivo experiments [33,43]. As compared to liposome-mediated delivery without BBBD, the doxorubicin concentration at 2 h after sonication was 2.08 times higher when FUS and MB were applied. This finding agrees with the animal experiments where FUS-MB-induced improvement was measured in the range of 1.51- to 2.65-fold [76]. Besides the BBBD model based on semi-empirical formulas from experiments [21,32], the drug transport model has been widely applied in previous numerical studies on drug delivery and validated by comparison with experimental results. The IFP and IFV were calculated as 1500 Pa [77] and 0.17 µm/s [12], respectively, which were within the experimental ranges of 586 to 4200 Pa [78] and 0.13 to 0.20 µm/s [79]. Model-predicted drug transport profiles were well fit to the measurements reported from ex vivo experiments, with the coefficient of multiple determination reaching 0.83 [80]. Although comparisons to animal experiments remain qualitative for small molecular drugs [81,82], the prediction accuracy can be largely improved for the drug vehicles in nanoscale [83]. Therefore, since this study was focused on drug transport rather than model development, the work of model validation was not duplicated here.

The present study offers some new insight into the enhancement of liposome-mediated drug delivery into brain tumour via FUS and MB; however, there were several assumptions involved. (I) FUS sonication is usually performed using an ultrasound transducer with its focus point swapping across the brain tumour, so the BBBD could be non-uniform across the entire brain. As the optimisation of FUS trajectory for achieving homogeneous BBBD was beyond the scope of this study, the enhancement of drug transvascular permeability was assumed to be perfectly restricted within the brain tumour and uniformly distributed. (II) Liposomes are able to cross the cell membrane by endocytosis and then release the drug intracellularly [84]. However, as with the liposomes used in study, polyethylene glycol (PEG) is usually applied to modify the liposome surface in order to achieve extensive survival time in blood circulation. This ligand effectively inhibits endocytosis by forming a steric barrier to prevent liposome–cell membrane interaction [85,86]. Therefore, endocytosis was not considered here, and liposomes were assumed to be impermeable to cell membrane. (III) The spatial distribution of microvasculature can be highly heterogeneous in large tumours. This heterogeneity can affect local drug supply, and thereby influence drug transport and accumulation. The microvasculature was assumed to be homogeneously distributed in the brain tumour, as there was a lack of relevant information that could be obtained from available medical images. This assumption could be relaxed by using dynamic, contrast-enhanced MR images [81], from which vasculature density can be predicted based on the time course of signal intensity at each image pixel.

It is of note that the mathematical model was developed to cover the key biophysical and physicochemical processes in drug delivery. The applied model parameters refer to the representative values that were extracted from the literature. The model predictions could be used for qualitative comparisons to examine the effects of specified processes, so as to provide guidance for the optimisation of treatment regimens and liposome properties. The modelling accuracy could be improved by employing patient-specific information and developing mathematical descriptions for particular processes in drug delivery. These would require extensive support from medical imaging and in vivo experiments, respectively.

## 5. Conclusions

Drug transport in the liposome-mediated delivery coupled with FUS- and MB-induced BBBD was investigated by means of numerical simulation in this study. A 3D brain tumour model reconstructed from MR images was applied with the aim of capturing the realistic geometrical characteristics of the lesion. Although doxorubicin could enter the tumour ECS when the BBB was disrupted by FUS and MB, modelling predictions demonstrated that the use of liposomes could further improve the treatment efficacy under the same delivery conditions. This improvement mainly relied on the enhanced transvascular permeability of free doxorubicin and the sustainable drug supply from the long-circulating liposomes when burst FUS sonication was applied. The anticancer effectiveness of this treatment could be improved by extending the sonication time window, which largely improved the accumulation of both liposomal and free drug in tumour ECS. The release rate needs to be optimised to achieve an acceptable trade-off among drug release, transvascular exchange and elimination. The BBBD is recommended to be carried out simultaneously with the bolus injection of liposomes, as late BBBD reduces the anticancer efficacy but retains a similar risk of cardiotoxicity. Results obtained from this study could provide suggestions for the future optimisation of FUS-MB–liposome-mediated drug delivery.

## Figures and Tables

**Table 1:** Parameters for chemotherapeutic drugs *.

Symbol	Parameter	Unit	Liposome	Doxorubicin
*P_ICS-ECS_*	Partition coefficient between ICS and ECS	-	-	1.0 [33]
*P_CM-ECS_*	Partition coefficient between CM and ECS	-	-	0.3 [34]
*K_IVS_*, *K_ECS_*, *K_ICS_*	Binding constant in IVS, ECS and ICS	-	-	3.0 [35]
*D_ECS_*	Diffusion coefficient in tissue ECS	m^2^/s	9.0 × 10^−12^ (T) [36] 5.8 × 10^−12^ (N) [36]	3.4 × 10^−10^ (T) [17] 1.6 × 10^−10^ (N) [17]
*P_0_*	Transvascular permeability with BBBD	m/s	3.4 × 10^−9^ (T) [29] 0.0 (N)	0.0 (T) 0.0 (N)
*σ*	Drug osmotic reflection coefficient	-	0.95 (T) [37] 1.0 (N) [37]	0.15 (T) [17] 0.15 (N) [17]
*k_e_*	Drug elimination rate in tissue	s^−1^	-	5.8 × 10^−4^ [17]
*k_c_*	Drug clearance rate in blood	s^−1^	3.9 × 10^−6^ [38]	2.4 × 10^−3^ [39]
*k_rel_*	Drug release rate from liposomes	s^−1^	1.0 × 10^−4^ [40]	-
*V_d_*	Distribution volume	m^3^	6.4 × 10^−3^ [36]	7.7 × 10^−3^ [39]

* T and N refer to the brain tumour and normal brain tissue, respectively.

**Table 2:** Parameters for the brain tumour and normal tissue.

Symbol	Parameter	Unit	Brain Tumour	Normal Tissue
*α*	Volume fraction of ECS	-	0.35 [41]	0.20 [33]
*β*	Volume fraction of ICS	-	0.55 [41]	0.65 [33]
*ρ*	Density of interstitial fluid	kg/m^3^	1.0 × 10^3^ [42]	1.0 × 10^3^ [42]
*μ*	Viscosity of interstitial fluid	kg/m/s	7.8 × 10^−4^ [42]	7.8 × 10^−4^ [42]
*π_b_*	Osmotic pressure of blood	Pa	3.4 × 10^3^ [43]	3.4 × 10^3^ [43]
*π_i_*	Osmotic pressure of interstitial fluid	Pa	1.1 × 10^3^ [12]	7.4 × 10^2^ [12]
*p_b_*	Pressure in intravascular space	Pa	4.6 × 10^3^ [43]	4.6 × 10^3^ [43]
*S/V*	Area of blood vessel surface per tissue volume	m^−1^	2.0 × 10^4^ [12]	7.0 × 10^3^ [12]
*σ_T_*	Osmotic reflection coefficient of tissue	-	0.82 [12]	0.91 [12]
*K_b_*	Hydraulic conductivity of blood vessel wall	m/Pa/s	1.1 × 10^−12^ [16]	1.4 × 10^−13^ [16]
*κ*	Tissue Darctian permeability	m^2^	6.4 × 10^−14^ [16]	6.5 × 10^−15^ [16]

**Table 3:** Predicted interstitial fluid flow.

Tissue Type	$IFP_{avg}\;(\mathrm{Pa})$	$IFV_{avg}\;(\mu m/s)$
Brain tumour	1071.79	0.43
Normal tissue	876.03	0.13

**Table 4:** *AUC*_24h_ (mg/mL·s) with different delivery modes.

Delivery Mode	IVS	Tumour ECS	Normal Tissue ECS
Direct administration + BBBD	3.47	1.37	2.54 × 10^−3^
Liposomes	17.78	9.68	1.07 × 10^−2^
Liposomes + BBBD	17.78	12.95	1.79 × 10^−2^

**Table 5:** *AUC*_24h_ (mg/mL·s) of the treatments using liposomes with different release rates.

Release Rate (s^−1^)	IVS	Tumour ECS	Normal Tissue ECS
*k_rel_* = 1.0 × 10^−6^	1.28 × 10^−3^	1.39 × 10^−3^	3.83 × 10^−6^
*k_rel_* = 1.0 × 10^−5^	9.20 × 10^−3^	9.69 × 10^−3^	2.44 × 10^−5^
*k_rel_* = 1.0 × 10^−4^	1.78 × 10^−2^	1.29 × 10^−2^	1.79 × 10^−5^
*k_rel_* = 1.0 × 10^−3^	1.83 × 10^−2^	7.98 × 10^−3^	9.96 × 10^−6^

**Table 6:** *AUC*_24h_ (mg/mL·s) of the treatments with different BBBD timings.

Delay (min)	IVS	Tumour ECS	Normal Tissue ECS
0 min	1.78 × 10^−2^	1.29 × 10^−2^	1.79 × 10^−5^
30 min	1.78 × 10^−2^	1.27 × 10^−2^	1.77 × 10^−5^
60 min	1.78 × 10^−2^	1.19 × 10^−2^	1.73 × 10^−5^
90 min	1.78 × 10^−2^	1.12 × 10^−2^	1.69 × 10^−5^

**Table 7:** *AUC*_24h_ (mg/mL∙s) of the treatments with different sonication durations.

Sonication Duration (min)	IVS	Tumour ECS	Normal Tissue ECS
Burst	1.78 × 10^−2^	1.29 × 10^−2^	1.79 × 10^−5^
15 min	1.78 × 10^−2^	1.48 × 10^−2^	2.02 × 10^−5^
30 min	1.78 × 10^−2^	1.61 × 10^−2^	2.19 × 10^−5^
45 min	1.78 × 10^−2^	1.70 × 10^−2^	2.31 × 10^−5^
